# Progressive multiple sequence alignment with indel evolution

**DOI:** 10.1186/s12859-018-2357-1

**Published:** 2018-09-21

**Authors:** Massimo Maiolo, Xiaolei Zhang, Manuel Gil, Maria Anisimova

**Affiliations:** 1 Institute of Applied Simulation, School of Life Sciences and Facility Management, Zurich University of Applied Sciences (ZHAW), Grüentalstrasse 14, P.O. Box, Waedenswil, CH-8820 Switzerland; 2 Institute of Molecular Life Sciences, University of Zurich, Winterthurerstrasse 190, Zurich, CH-8057 Switzerland; 3 National Heart and Lung Institute, Imperial College London, South Kensington Campus, London, SW7 2AZ UK; 4 Swiss Institute of Bioinformatics (SIB), Quartier Sorge - Bâtiment Génopode, Lausanne, CH-1015 Switzerland

**Keywords:** Sequence alignment, Indel, Phylogeny, Dynamic programming, Poisson process

## Abstract

**Background:**

Sequence alignment is crucial in genomics studies. However, optimal multiple sequence alignment (MSA) is NP-hard. Thus, modern MSA methods employ progressive heuristics, breaking the problem into a series of pairwise alignments guided by a phylogeny. Changes between homologous characters are typically modelled by a Markov substitution model. In contrast, the dynamics of indels are not modelled explicitly, because the computation of the marginal likelihood under such models has exponential time complexity in the number of taxa. But the failure to model indel evolution may lead to artificially short alignments due to biased indel placement, inconsistent with phylogenetic relationship.

**Results:**

Recently, the classical indel model TKF91 was modified to describe indel evolution on a phylogeny via a Poisson process, termed PIP. PIP allows to compute the joint marginal probability of an MSA and a tree in linear time. We present a new dynamic programming algorithm to align two MSAs –represented by the underlying homology paths– by full maximum likelihood under PIP in polynomial time, and apply it progressively along a guide tree. We have corroborated the correctness of our method by simulation, and compared it with competitive methods on an illustrative real dataset.

**Conclusions:**

Our MSA method is the first polynomial time progressive aligner with a rigorous mathematical formulation of indel evolution. The new method infers phylogenetically meaningful gap patterns alternative to the popular PRANK, while producing alignments of similar length. Moreover, the inferred gap patterns agree with what was predicted qualitatively by previous studies. The algorithm is implemented in a standalone C++ program: https://github.com/acg-team/ProPIP. Supplementary data are available at BMC Bioinformatics online.

**Electronic supplementary material:**

The online version of this article (10.1186/s12859-018-2357-1) contains supplementary material, which is available to authorized users.

## Background

Multiple sequence alignments (MSAs) are routinely required in the early stages of comparative and evolutionary genomics studies. Not surprisingly, accuracy of MSA inference affects subsequent analyses that rely on MSA estimates [1]. MSA estimation is among the oldest bioinformatics problems, yet remains intensely studied due to its complexity (NP-hard [2–4]). The progressive alignment approach has allowed to reduce the overall computational complexity to polynomial time by breaking the MSA problem into a series of pairwise alignments guided by a tree representing the evolutionary relationship of sequences. Today most popular alignment programs employ the progressive approach (e.g., ClustalW [5], MAFFT [6], MUSCLE [7], PRANK [8, 9] and T-Coffee [10] among others).

All state-of-the-art MSA programs nowadays use an evolutionary model to describe changes between homologous characters, providing a more realistic description of molecular data and thus more accurate inferences. However, a mathematical formulation of the insertion-deletion (indel) process still remains a critical issue. Describing the indel process in probabilistic terms is more challenging: unlike substitutions, indels often involve several sites, vary in length and may overlap obscuring the underlying mechanisms. Instead, the popular PRANK program adopts a pragmatic approach; it uses an outgroup to distinguish insertions from deletions during the progressive alignment procedure, so that insertions are not over-penalized [9]. As a result, PRANK produces exceptionally accurate alignments, notably with densely sampled data and given an accurate guide tree. Still, the method lacks a mathematical model describing the evolution of indels. Indeed, the computation of the marginal likelihood under the classical indel models TKF91 [11] and TKF92 [12] is exponential in the number of taxa due to the absence of site independence assumption.

A recent modification of TKF91 describes the evolution of indels on a phylogenetic tree as a Poisson process, thus dubbed the Poisson indel process or the PIP model [13]. The indels occur uniformly within a sequence. Standard mathematical results, particularly the Poisson thinning, allow achieving linear time complexity for computing the joint marginal probability of a tree and an MSA. This includes analytic marginalisation of unobservable homologous paths which occur whenever an ancestral character is inserted and subsequently deleted, and consequently cannot be detected in the extant sequences. For a given MSA and a tree, a likelihood score under PIP can be computed in linear time. This score can be used to find the maximum a posteriori tree-alignment solution. Remarkably, this breakthrough allows for a necessary rigorous way of combining models of substitutions and indels, and a tractable computation of the marginal likelihood function. At the moment the algorithm has only been applied in a Bayesian framework via tree-alignment space sampling.

Here we propose a new progressive algorithm to estimate an MSA under the explicit model of substitutions and indels. We have re-framed the original PIP equations into a dynamic programming (DP) approach. It aligns two MSAs –represented by their homology paths on the two corresponding subtrees– by maximum likelihood (ML) in polynomial time. The progressive algorithm traverses a guide tree in postorder; at each internal node the DP is applied to align the two sub-alignments at the child nodes. The procedure terminates at the root of the guide tree, with the complete MSA and the corresponding likelihood, which by construction is the likelihood under the PIP model. We have implemented the progressive MSA algorithm in a prototype program and verified its correctness by simulation. To our knowledge, this is the first progressive MSA algorithm with polynomial time complexity, using a mathematical formulation of an explicit indel process. Note that an equivalent formulation under TKF91 or TKF92 –i.e. using the full marginal likelihood along the subtrees in question– would have exponential time complexity. Quadratic time complexity under the TKF models could be obtained [14] by representing sequences at internal nodes through probability profiles, and aligning those. However, this approach does not consider the evolutionary history in the subtrees.

The remainder of this manuscript is organized as follows. We first introduce notation and the PIP model. Then, we describe our DP algorithm and provide the simulation results. We conclude the paper with an illustrative real dataset, where we contrast our method with PRANK, as well as with MAFFT, representing a state of the art similarity based progressive method.

## Methods

### Preliminaries: the PIP model

Let $\tau = (\mathcal {V}, \mathcal {E}, b)$ represent a rooted binary phylogenetic tree with *N* leaves. *τ* is a directed, connected, labelled acyclic graph, with a finite set of branching points $\mathcal {V}$ of cardinality $|\mathcal {V}|=2N-1$ and a set of edges $\mathcal {E} \subset \mathcal {V} \times \mathcal {V}$. Leaves $\mathcal {L} \subset \mathcal {V}$ denotes *N* observed taxa, represented by strings of characters from a finite alphabet *Σ* (nucleotides, amino acids or codons). There are *N*−1 internal vertices $v \subset \mathcal {V}$ whereof the root *Ω* is the most recent common ancestor of all leaves. Branch length *b*(*v*) associated with node $v \in \mathcal {V}$ spans from *v* to its parent node pa (*v*). The total tree length ∥*τ*∥ is a sum of all the branch lengths.

The PIP model describes a string-valued evolutionary process along the branches of *τ*. We denote the distance from the root to a given point on the tree, by the same symbol *τ*. Atomic insertions are Poisson events with rate measure *ν*(d*t*)=*λ*(*τ*(d*t*)+*μ*^−1^*δ*_*Ω*_(d*t*)), where *λ* is the insertion rate, *μ* the deletion rate, and *δ*_*Ω*_(·) Dirac’s delta function. This formulation guarantees that the expected sequence length remains constant during the whole evolutionary process. Point substitutions and deletions are modelled by a continuous-time Markov process on *Σ*_*ε*_=*Σ*∪{*ε*}, where *ε* is the deletion symbol. Accordingly, the generator matrix **Q**_*ε*_ of the combined substitution and indel process extends the instantaneous substitution rate matrix **Q** by a row and a column to include *ε*, which is modelled as an absorbing state as there can be no substitutions after a deletion event. The quasi-stationary distribution of **Q**_*ε*_ is denoted by ***π***_*ε*_. Root *Ω* has a virtual infinite length stem, reflecting the equilibrium steady state distribution at the root.

For an internal node *v*, the probability *ι*(*v*) of inserting a single character on branch pa (*v*)→*v*, is proportional to branch length *b*(*v*). For *v*≠*Ω* it is given by *ι*(*v*)=*b*(*v*)/(∥*τ*∥+*μ*^−1^); at the root atomic mass point probability *ι*(*Ω*)=*μ*^−1^/(∥*τ*∥+*μ*^−1^) so that ${\sum \nolimits }_{v \in \mathcal {V}}\iota (v)=1$. The survival probability *β*(*v*) associated with an inserted character on branch pa (*v*)→*v* is given by *β*(*Ω*)=1 and *β*(*v*)=(1− exp(−*μb*(*v*)))/(*μb*(*v*)).

The marginal likelihood *p*_*τ*_(*m*) of MSA *m* of length |*m*| is computable in *O*(*N*·|*m*|) and can be expressed as

1 $$ p_{\tau}(m) = \varphi(p(c_{\emptyset}), |m|) \prod_{c\in m} p(c),  $$

where *p*(*c*) is the likelihood of a single column *c*, and *p*(*c*_*∅*_) is the likelihood of an unobservable character history, represented by a column *c*_*∅*_ with a gap at every leaf. The factor in (1)

2 $$ \varphi(p(c_{\emptyset}), |m|)=\|\nu\|^{|m|}\exp\left(\|\nu\| \left(p(c_{\emptyset})-1 \right)\right)/|m|!  $$

is the marginal likelihood over all unobservable character histories, where ∥*ν*∥ is the normalising Poisson intensity.

The column likelihood can be expressed as

3 $$ p(c) = \sum\limits_{v \in \mathcal{V}}\iota(v)f_{v},  $$

where *f*_*v*_ denotes the probability of the homology path underlying column *c*, given that the corresponding character was inserted at *v*. This probability can be computed in *O*(*N*) using a variant of Felsenstein’s peeling recursion [15]. Let $\mathcal {S}$ be the set of leaves that do not have a gap in column *c*, and $\mathcal {A}$ the set of nodes ancestral to $\mathcal {S}$. Then

4 $$\begin{array}{*{20}l} f_{v} = \left\{ \begin{array}{l r} \mathbf{1}\left[ v\in \mathcal{A} \right] \beta(v){\sum\nolimits}_{\sigma\in\Sigma}\boldsymbol{\pi}_{\epsilon}(\sigma)\tilde{f}_{v}(\sigma)&\text{if \(c \neq c_{\emptyset}\)}\\ 1-\beta(v)+\beta(v){\sum\nolimits}_{\sigma\in\Sigma}\boldsymbol{\pi}_{\epsilon}(\sigma)\tilde{f}_{v}(\sigma)&\text{o.w.},\\ \end{array} \right. \end{array} $$

where

5 $$\begin{array}{*{20}l} \tilde{f}_{v}(\sigma)=\left\{ \begin{array}{l r} \mathbf{1}[c(v) = \sigma] \hfill \text{if} v \in \mathcal{L}\\ {\prod_{w\in\text{child}(v)}} \left[ {\sum\limits_{\sigma^{\prime} \in \Sigma_{\epsilon}}}{\exp(b(w)\mathbf{Q}_{\epsilon})_{\sigma,\sigma^{\prime}}} \tilde{f}_{w}(\sigma^{\prime}) \right] \hfill \text{o.w.},\\ \end{array} \right. \end{array} $$

and **1**[·] is the indicator function. In Eq. 4, the term 1−*β*(*v*) accounts for the probability that the inserted character does not survive till the first node below the insertion point. The recursive function $\tilde {f}_{v}$ computes the probability of the substitution-deletion process of a single character.

### Dynamic programming algorithm under PIP

Given an internal node *v*, our DP algorithm proceeds to align the two sub-alignments obtained in the left and right sub-trees maximizing the likelihood (Eq. 1) of the tree rooted at *v*. Let **X** and **Y** denote these sub-alignments, respectively with *N*_**X**_ and *N*_**Y**_ sequences and alignment lengths |**X**| and |**Y**|. If a sub-tree is a leaf then the sub-alignment, say **X**, is reduced to an input sequence, i.e. *N*_**X**_=1 and |**X**| corresponds to the sequence length.

**Fig. 1:**
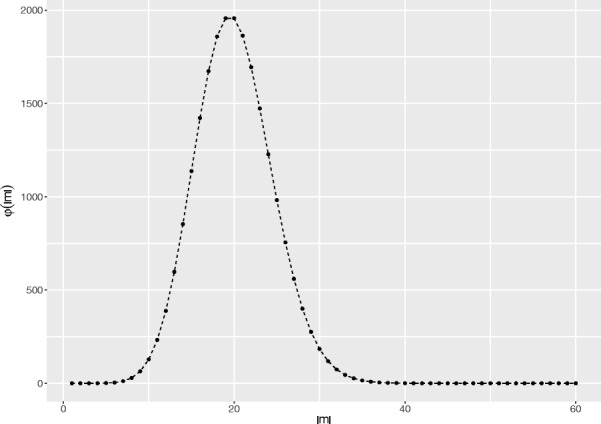
An example of *φ*(|*m*|) (Eq. 2), i.e. the marginal likelihood of all non-observable histories, as a function of MSA length |*m*|. The parameters are: *τ*=1, *λ*=10, *μ*=1, *p*(*c*_*∅*_)=0.5

Note that the marginal likelihood function *p*_*τ*_(*m*) (Eq. 1) is not monotonically increasing in the alignment length |*m*|. While the product of column likelihoods is monotonically increasing, the marginal likelihood of unobserved histories *φ*(*p*(*c*_*∅*_),|*m*|) is non-monotonic (Fig. 1). This means that *p*_*τ*_(*m*) cannot be maximised by means of a standard two-dimensional DP approach (in particular, because the alignment length is not known a priori). Similarly to TKF91 [11], we need three DP matrices, one for each state (i.e. match, gapX and gapY), however to account for the dependence on alignment length we have extended the matrices with a third dimension.

The algorithm works with three three-dimensional sparse matrices **S**^M^, **S**^X^ and **S**^Y^ each of size (|**X**|+1)×(|**Y**|+1)×(|**X**|+|**Y**|+1) with entries defined as follows (Fig. 2b):

1. *match* cell $\mathrm {\mathbf {S}}^{\mathrm {M}}_{i,j,k}$ contains the likelihood of the partial optimal MSA of length *k* between **X**_1_…**X**_*i*_ and **Y**_1_…**Y**_*j*_ with the columns **X**_*i*_ and **Y**_*j*_ aligned. Consequently, all characters in the two columns are inferred to be homologous.

**Fig. 2:**
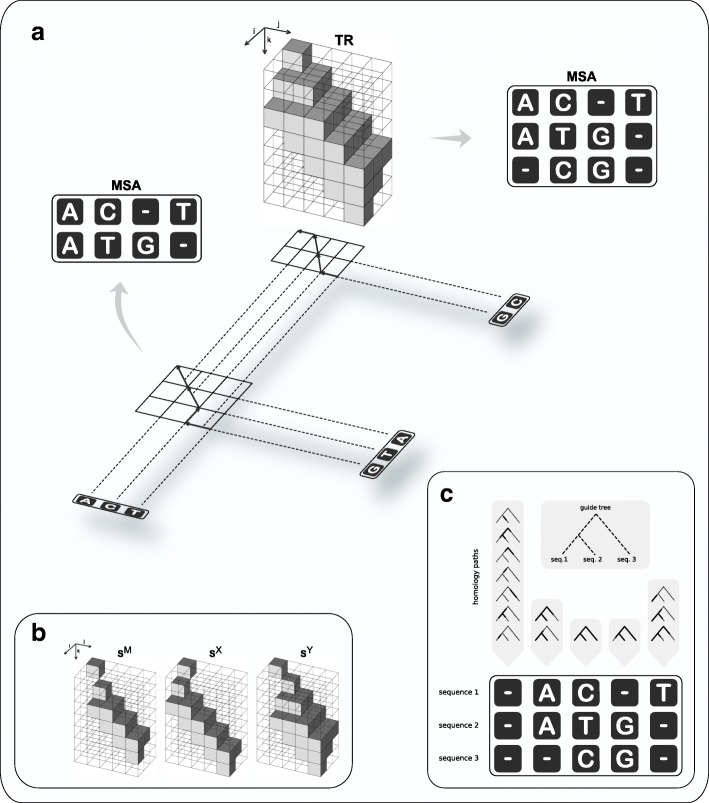
Overview of the progressive algorithm. The algorithm traverses a guide tree (indicated by the shadow in Panel **a**) in postorder. At each internal node, the evolutionary paths from the two children down to the leaves (doted lines in Panel **a**) are aligned by full maximum likelihood under the PIP model, using a dynamic programming approach (DP). Since the likelihood function does not increase monotonically in the MSA length (see Fig. 1), the DP accommodates the MSA length along a third dimension (indicated by *k* in Panels **a**, **b**); thus, it works with cubic matrices (in contrast to the traditional quadratic DP alignment). The forward phase of the DP stores likelihood values in three sparse matrices (Panel **b**: **S**^M^ for matching columns; **S**^X^ and **S**^Y^ to introduce new indel events). Further, matrix **TR** (Panel **a**) at position (*i*,*j*,*k*) records the name of the DP matrix (either “ **S**^M^”, “ **S**^X^”, or “ **S**^Y^”) with highest likelihood at (*i*,*j*,*k*). An optimal alignment is determined by backtracking along **TR** (indicated in Panel **a** by the arrows in the projection of **TR** onto the plane). Note that the likelihood function marginalises over all indel scenarios compatible with putative homology (Panel **c**)
2. *gapX* cell $\mathrm {\mathbf {S}}^{\mathrm {X}}_{i,j,k}$ contains the likelihood of the partial optimal MSA of length *k* between **X**_1_…**X**_*i*_ and **Y**_1_…**Y**_*j*_ with the column **X**_*i*_ aligned with a column of size *N*_**Y**_ containing gaps only. The characters in the two columns do not share a common history, either because the ancestor character had been deleted on the right subtree, or because it had been inserted on the left subtree, below the node *v*.
3. similarly, *gapY* cell $\mathrm {\mathbf {S}}^{\mathrm {Y}}_{i,j,k}$ matches column **Y**_*j*_ with a column of size *N*_**X**_ containing gaps only.

### Forward phase

Each matrix **S**^M^, **S**^X^ and **S**^Y^ is initialized with *φ*(*p*(*c*_*∅*_)),0) at position (0,0,0) and a zero in every other position. The DP equations are:

6 $$\begin{array}{*{20}l} \mathrm{\mathbf{S}}^{\mathrm{M}}_{i,j,k}= \frac{\|\nu\|}{k} \cdot p\left(\left[\begin{array}{l}\mathrm{\mathbf{X}}_{i}\\ \mathrm{\mathbf{Y}}_{j}\end{array}\right]\right) \cdot\text{max}\Big\{\Big. &\mathrm{\mathbf{S}}^{\mathrm{M}}_{i-1,j-1,k-1}, \\ &\mathrm{\mathbf{S}}^{\mathrm{X}}_{i-1,j-1,k-1}, \\ &\mathrm{\mathbf{S}}^{\mathrm{Y}}_{i-1,j-1,k-1} \Big\}\Big. \end{array} $$

7 $$\begin{array}{*{20}l}\mathrm{\mathbf{S}}^{\mathrm{X}}_{i,j,k}= \frac{\|\nu\|}{k} \cdot p\left(\left[\begin{array}{l}\mathrm{\mathbf{X}}_{i}\\c_{\emptyset}\end{array}\right]\right) \cdot\text{max}\Big\{\Big. &\mathrm{\mathbf{S}}^{\mathrm{M}}_{i-1,j,k-1},  \\ &\mathrm{\mathbf{S}}^{\mathrm{X}}_{i-1,j,k-1}, \\ &\mathrm{\mathbf{S}}^{\mathrm{Y}}_{i-1,j,k-1}\Big\}\Big. \end{array} $$

8 $$\begin{array}{*{20}l} \mathrm{\mathbf{S}}^{\mathrm{Y}}_{i,j,k}=\frac{\|\nu\|}{k} \cdot p\left(\left[\begin{array}{l}c_{\emptyset}\\\mathrm{\mathbf{Y}}_{j}\end{array}\right]\right) \cdot\text{max}\Big\{\Big. &\mathrm{\mathbf{S}}^{\mathrm{M}}_{i,j-1,k-1}, \\ &\mathrm{\mathbf{S}}^{\mathrm{X}}_{i,j-1,k-1}, \\ &\mathrm{\mathbf{S}}^{\mathrm{Y}}_{i,j-1,k-1} \Big\}\Big. \end{array} $$

$${}\text{for} \ i=1,\ldots,|\mathrm{\mathbf{X}}|, j=1,\ldots,|\mathrm{\mathbf{Y}}|\text{ and} k=1,\ldots,|\mathrm{\mathbf{X}}|+|\mathrm{\mathbf{Y}}|. $$

The symbol *c*_*∅*_ in Eqs. 7 and 8 represents a column with gaps, respectively of length *N*_**Y**_ and *N*_**X**_. The factor ∥*ν*∥/*k* successively constructs *φ*(*p*(*c*_*∅*_),*k*) along the third dimension as columns are added into partial alignments.

As pointed out above, a column likelihood under PIP (Eq. 1) can be computed recursively in linear time in the number of input sequences. The recursion corresponds to a postorder tree traversal (Eq. 5), which coincides with the tree traversal of our progressive algorithm. As a consequence, during the progressive alignment a column likelihood for the DP (*p*(·) in Eqs. 6–8) at a particular node *v* can be computed in constant time by re-using appropriate summands (defined by Eq. 4) from the column likelihoods at the two children of *v*. In particular, the set $\mathcal {A}$ can be constructed from the corresponding sets at the two children $\mathcal {A}_{\text {left}}$ and $\mathcal {A}_{\text {right}}$:

9 $$\begin{array}{*{20}l} \mathcal{A} = \left\{ \begin{array}{l l} \left\{ v \right\rbrace&\text{for match state}\\ \mathcal{A}_{\text{left}} \,\cup\, \left\{ v \right\rbrace&\text{for gapX state}\\ \mathcal{A}_{\text{right}} \,\cup\, \left\{ v \right\rbrace&\text{for gapY state}\\ \end{array} \right. \end{array} $$

Consequently, the total asymptotic running time of the forward phase is *O*(*Nl*^3^), where *l* is bounded by the length of the longest input sequence. The independence structure of the DP along the dimension of the MSA length (i.e. index *k*) readily allows parallelisation; all the entries in the DP matrices for a fixed *k* can be computed in parallel from the entries at the layer *k*−1, taking down the time to *O*(*Nl*).

### Backtracking

An optimal alignment is determined by backtracking along a trace-back matrix **TR** of size (|**X**|+1)×(|**Y**|+1)×(|**X**|+|**Y**|+1). In the forward phase, **TR** records at position (*i*,*j*,*k*) the name of the DP matrix (either “ **S**^M^”, “ **S**^X^”, or “ **S**^Y^”) with highest likelihood at the same position (*i*,*j*,*k*). If the maximum is not unique then a uniform random choice is made. The backtracking algorithm starts at **TR**(|**X**|,|**Y**|,*k*_0_), where

$$\begin{array}{*{20}l} k_{0}=arg\,max_{\substack{k = \text{max}(|\mathrm{\mathbf{X}}|,|\mathrm{\mathbf{Y}}|)\ldots(|\mathrm{\mathbf{X}}|+|\mathrm{\mathbf{Y}}|) }}s(k) \end{array} $$

with

$$\begin{array}{*{20}l} s(k)=\Big\{\Big. &\mathrm{\mathbf{S}}^{\mathrm{M}}(|\mathrm{\mathbf{X}}|,|\mathrm{\mathbf{Y}}|,k), \mathrm{\mathbf{S}}^{\mathrm{X}}(|\mathrm{\mathbf{X}}|,|\mathrm{\mathbf{Y}}|,k), \mathrm{\mathbf{S}}^{\mathrm{Y}}(|\mathrm{\mathbf{X}}|,|\mathrm{\mathbf{Y}}|,k)\Big\}\Big. \end{array} $$

is the length of the best scoring alignment. If *k*_0_ is not unique a random uniform choice is made. **TR** is then traversed from (|**X**|,|**Y**|,*k*_0_) to (0,0,0). Suppose the algorithm is at position (*i*,*j*,*k*). If **TR**(*i*,*j*,*k*)= “ **S**^M^” then the columns **X**_*i*_ and **Y**_*j*_ are matched and all the indices are decremented, i.e. *i*←*i*−1, *j*←*j*−1, *k*←*k*−1. If **TR**(*i*,*j*,*k*) is set to “ **S**^X^” then the column **X**_*i*_ is matched with a column of gaps of size *N*_**Y**_ and the indices *i* and *k* are decremented, and, if **TR**(*i*,*j*,*k*) contains the value “ **S**^Y^” then the column **Y**_*j*_ is matched with a column of gaps of size *N*_**X**_ and the indices *j* and *k* are decremented.

## Results

Since the main goal of the article is to describe a new method, it is desirable to evaluate the correctness of the implementation (i.e., likelihood values and optimisation) and the accuracy of the estimator. Correctness can be evaluated by simulations under the true model or by comparison with existing implementations. The evaluation of alignment accuracy is more problematic ([16]), because the historical evolutionary events are not observable, so that we have no access to true alignments. Benchmarks like BAliBASE have attempted to provide sets of reference alignments. Those, however, represent structural similarity, not necessarily reflecting homology, but also could be due to structural convergence. Moreover, benchmarks tend to represent alignments with highly compact and conserved cores offering little information about indel placement ([16]). Alternatively, synthetic data can be generated, where the true alignments are known. However, simulations rely on a generative model, which never perfectly correspond to the real process. The closer the generative model is to the assumed by the estimator, the better the estimator should perform.

Recently, it has been shown that the results obtained from structural benchmarks and from phylogenetic simulations have produced inconsistent results ([17–20]). Phylogeny-aware aligners like PRANK tend to perform well in simulations, while poorly on structural benchmarks. This can be explained by the fact that the objective of phylogenetic aligners is to infer evolutionary homology, rather than conserved structural features.

Below we provide results from some basic evaluations of our proposed method.

### Empirical verification of correctness

To test the correctness of the algorithm and implementation, we generated data under PIP using a simulator provided by the authors of PIP. We chose relatively small trees and short sequences to be able to perform analytical tests during algorithm design and program debugging. Specifically, we simulated 120 datasets in total, on trees with 4, 5, 6 and 7 leaves, and using the following parameter combinations (*λ*,*μ*)∈{(0.1,0.1),(0.1,1),(1,0.1),(1,1)}. The resulting sequence lengths varied between 5 and 8 nucleotides.

First, we confirmed the correctness of the likelihoods obtained with the DP algorithm, by scoring the resulting MSAs with an independent implementation provided by the authors of PIP. In all cases the likelihoods matched. In a second test, we verified that the DP generates optimal pairwise MSA alignments. To this end, all the possible pairwise alignments were generated at each internal node of the guide-trees and scored with the independent implementation. The DP algorithm always reconstructed an optimal MSA.

### Aligning simulated data

To assess the quality of inferred alignments we have applied our method to simulated data that was previously used to evaluate PRANK [8]). These data sets were each 1000 nucleotides long and were generated under realistic evolutionary parameters on 16- 32- and 64-taxon trees and with different degrees of divergence. Note that indel lengths were drawn from a Poisson distribution with a mean of 1.7 bases. Inferred MSA lengths and four standard quality scores obtained with our method were compared to those inferred with MAFFT v7.402 (with option –auto) and PRANK v.140603 (with options -protein -termgap -nomissing -once, with and without the +F option). The results of this comparison are shown in Additional file 1: Table S1 and Figure S1. No matter what evaluation score was considered, progressive alignment under PIP produced alignment quality similar to both PRANK and MAFFT. In terms of approaching the true MSA length, our method infers alignments of a similar length to PRANK but consistently outperform MAFFT. In many cases, our method also infers MSA lengths closer to the true compared to PRANK, albeit by a small margin. These results are encouraging, especially considering that the simulation scenario with long indels explicitly favours MAFFT and PRANK, both of which allow for long indels in their scoring schemes, although they are not explicitly modelled.

### Aligning sequences from HIV/SIV envelope glycoprotein gp120

**Fig. 3:**
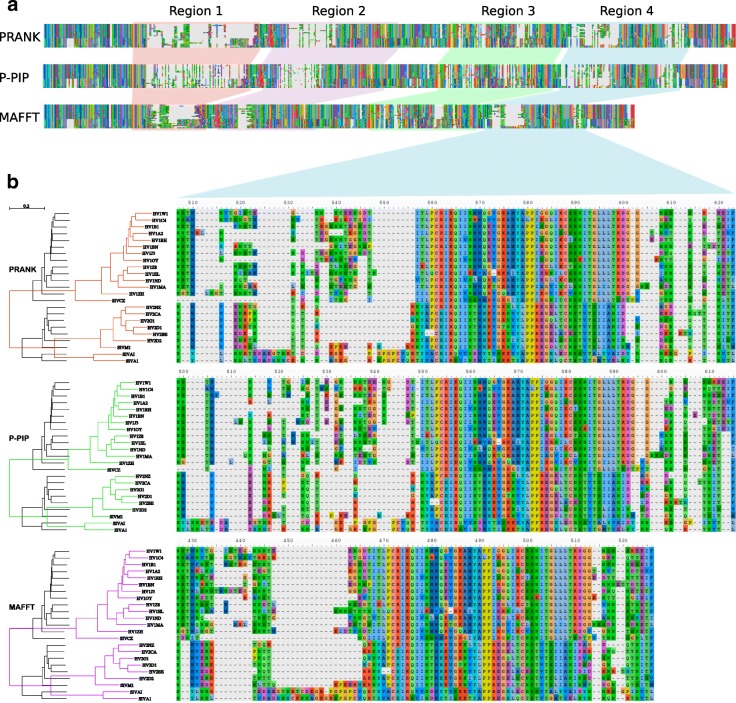
MSAs inferred with PRANK+F (top), our algorithm (middle, denoted by P-PIP) and MAFFT (bottom) from 23 strains of gp120 human and simian immunodeficiency virus (always using the same guide-tree). **a**. The total MSA lengths are 669, 661 and 579 columns respectively. The three methods show good agreement in the conserved regions. Substantial differences are observed in regions 1–4, highlighted by colors. **b**. Magnification of Region 4. MAFFT over-aligns the sequences. Depicted on the left: The tree in black is the original guide-tree. The trees depicted in colour are the same guide tree but with re-estimated branch lengths. A detailed view of regions 1–3 is given in Additional file 1: Figures S1-S3

Using our new algorithm we inferred an MSA for a challenging dataset, 23 envelope glycoprotein gp120 sequences from HIV/SIV, previous analysed by Löytynoja and Goldman [8]. We compared the results of our algorithm with the MSAs inferred by MAFFT and PRANK. The resulting MSAs (Fig. 3) showed good agreement in the conserved regions. Indeed, the use of structural benchmarks [16], which are mainly restricted to such regions, has illustrated that it is difficult to distinguish state-of-the-art aligners. In contrast, variable regions display distinctly different indel patterns, which was reflected in the MSA lengths. Consistent with previous reports [8, 21] MAFFT over-aligns the sequences resulting in a short alignment (579 columns). The alignment inferred with our method had similar length (661 columns) to the one inferred by PRANK (669 columns).

The indel patterns reflected the underlying indel model or scoring function of the methods. Our algorithm favoured shorter indels, compared to PRANK and MAFFT, which reconstructed visually tidier gap regions. A phylogenetic interpretation of MAFFT’s indel placement implies few insertions, followed by several subsequent deletions, leading to a short MSA. PRANK infers a longer alignment, with phylogenetically meaningful and balanced number of insertions and deletions. Note that similar to MAFFT, PRANK also tends to block long indels. Our method infers a phylogenetically meaningful MSA, with multiple single amino-acid insertions, which sometimes fuse to mimic long indels (e.g., 4 amino-acids from #501 to #504). Our method infers short indels, which allows for gap regions with higher conservation in terms of the substitution rates; we observe more conserved columns. To quantify this, we estimated tree-lengths (in expected substitutions per site), by fitting the branch-lengths of the guide-tree topology based on the inferred MSAs using PhyML [22]. Consistent with the visual observation, our algorithm leads to the shortest tree (4.35), compared to PRANK (4.60) and MAFFT (4.90).

## Discussion

Here for the first time in the frequentist framework, we have developed and implemented a progressive MSA algorithm with an explicit evolutionary model of substitutions, insertions and deletions. The evolution of indels was described as a Poisson process as part of a continuous-time Markov model known as PIP. At the core of our method we have designed a new DP algorithm for the alignment of two MSAs by ML, which exploits PIP’s linear time complexity for the computation of marginal likelihoods. The overall complexity of the progressive algorithm is *O*(*Nl*^3^), where *N* is number of taxa and *l* is the maximum sequence length. The cubic factor stems from the fact that the likelihood is not monotonically increasing in the MSA length, so that the length has to be incorporated as an extra dimension in the DP. The *O*(*l*^2^) entries in a specific matrix layer along that dimension (i.e. corresponding to one particular alignment length) depend only on the layer above (and not on each other). Therefore, their computation can be parallelized, taking down the running time to *O*(*Nl*), assuming *O*(*l*^2^) processors. Further, our empirical findings show that the likelihood has exactly one maximum, suggesting an early stop condition to the DP. We are currently optimising our implementation with respect to this and other time-critical aspects. To date inference of MSAs under an evolutionary indel model (TKF91 or TKF92) has only been implement using a Bayesian framework. Such approaches are however computationally expensive with large datasets. Our method for MSA inference under PIP is the first step towards equivalent developments in the frequentist framework.

Despite only allowing single residue indels our method appears to fare surprisingly well compared to other state-of-the-art popular alignment tools such as PRANK and MAFFT. Indeed, our example above (as well as other preliminary data analyses, not shown) demonstrate that our new method allows inferring alignments with phylogenetically sensible gap patterns, similar to the phylogenetically-aware PRANK. In contrast to traditional aligners that do not utilise phylogenetic information to distinguish insertions and deletions, our method produces longer alignments, avoiding the artificial compression of MSAs and inferring more indels, again similar to PRANK. According to the underlying indel model, our method appears to infer more shorter indels (e.g., compared to PRANK and MAFFT), while longer indels are described by several subsequent indel events. Including longer indels is considered desirable, however it has not been studied whether modeling one residue indels at a time may also work well. For example, for simplicity models of codon substitution typically allow only one-nucleotide mutations. Despite this gross simplification codon models have been demonstrated to perform extremely well for practical analyses of protein-coding genes. As can be seen in our example of an HIV protein gp120, it is unclear what inferred indel pattern is more realistic (given that alignments inferred by our methods and by PRANK have very similar length). Considering the nature of HIV mutations, it is quite plausible that indel evolution of gp120 is dominated by short indel events [23]. Arguably, in our example, indel penalisation of PRANK and MAFFT (affine penalty schemes allowing for long indels) might make these tools too restrictive to single-residue indels, leading to aesthetically more pleasing alignments. PIP might be more restrictive to long indels but also more realistic for sequence data dominated by short indel events. Both alignment benchmarking and the parameter optimisation of gap penalties are extremely difficult due to the absence of sufficiently challenging datasets where true alignments are known.

## Conclusion

Our new methods provides not only a first step towards the explicit modeling of indels in the frequentist framework but also enables to test a different hypothesis of indel evolution. In our follow up studies we intend to further scrutinise the various properties of our new method, its further development including less greedy algorithm versions, variation of indel rates across sites and the approximations to include longer indels.

## Additional file

Additional file 1 Supplemental Materials. Qscores, MSA length and MSA magnifications. (PDF 3392 kb)

## Abbreviations

DP: Dynamic programming
Indel: Insertion and deletion
ML: Maximum likelihood
MSA: Multiple sequence alignment
PIP: Poisson indel process
