# Supplementary material for: Progressive multiple sequence alignment with indel evolution
Source: BMC Bioinformatics. 2018 Sep 21;19:331. doi: 10.1186/s12859-018-2357-1 (PMC6151001; doi:10.1186/s12859-018-2357-1)
Supplement: Supplementary file 1 — Supplemental Materials. Qscores, MSA length and MSA magnifications. (PDF 3392 kb) [file 12859_2018_2357_MOESM1_ESM.pdf]

		2X	4X	close	intermediate	distant
PRNK	Q	0.9932 (0.0020)	0.9926 (0.0020)	0.9928 (0.0024)	0.9754 (0.0058)	0.9354 (0.0128)
	TC	0.9547 (0.0092)	0.9271 (0.0120)	0.9696 (0.0086)	0.9080 (0.0177)	0.7931 (0.0317)
	Cline	0.9944 (0.0016)	0.9940 (0.0015)	0.9941 (0.0019)	0.9802 (0.0046)	0.9488 (0.0102)
	Modeler	0.9933 (0.0020)	0.9928 (0.0018)	0.9928 (0.0024)	0.9756 (0.0057)	0.9366 (0.0124)
PRNK+F	Q	0.9931 (0.0019)	0.9926 (0.0019)	0.9928 (0.0024)	0.9755 (0.0059)	0.9354 (0.0129)
	TC	0.9546 (0.0095)	0.9271 (0.0115)	0.9693 (0.0087)	0.9081 (0.0178)	0.7929 (0.0320)
	Cline	0.9944 (0.0016)	0.9940 (0.0015)	0.9941 (0.0019)	0.9803 (0.0046)	0.9488 (0.0102)
	Modeler	0.9932 (0.0019)	0.9928 (0.0018)	0.9928 (0.0024)	0.9756 (0.0058)	0.9366 (0.0124)
MFFT	Q	0.9896 (0.0037)	0.9895 (0.0028)	0.9889 (0.0043)	0.9606 (0.0099)	0.9010 (0.0181)
	TC	0.9355 (0.0154)	0.8975 (0.0182)	0.9551 (0.0144)	0.8623 (0.0270)	0.7033 (0.0398)
	Cline	0.9915 (0.0030)	0.9914 (0.0023)	0.9910 (0.0034)	0.9682 (0.0078)	0.9212 (0.0145)
	Modeler	0.9890 (0.0040)	0.9889 (0.0031)	0.9884 (0.0046)	0.9581 (0.0105)	0.8957 (0.0188)
P-PIP	Q	0.9891 (0.0028)	0.9892520 (0.0024)	0.9888 (0.0034)	0.9654 (0.0069)	0.9207 (0.0141)
	TC	0.9276 (0.0139)	0.88886800 (0.0153)	0.9525 (0.0124)	0.8686 (0.0215)	0.7386 (0.0329)
	Cline	0.9913 (0.00231)	0.99146400 (0.0019)	0.9911 (0.0027)	0.9728 (0.0055)	0.9380 (0.0113)
	Modeler	0.9891 (0.0029)	0.9891600 (0.0024)	0.9887 (0.0035)	0.9648 (0.0071)	0.9189 (0.0144)

Table S1 Quality scores. The alignments obtained with PRANK, PRANK+F, MAFFT and our Progressive-PIP have been compared using 5 different score metrics computed with Qscore [25]. The table reports the mean Q scores (SPS), TC (CS), Shift scores (Cline) and Modeler scores for the different evolutionary scenarios ('2X', '4X', 'close', 'intermediate', 'distant'), standard deviation in parentheses. The analyses were performed on the five scenarios simulated in [8]. The following type of MSAs were simulated: "close", "intermediate", "distant", "2X" and "4X", reflecting divergence levels. Each dataset contained 250 MSAs generated along a symmetric 16- (close, intermediate, distant), 32- (2X) and 64-taxon tree (4X). The branch lengths were 0.025 (close), 0.050 (intermediate), 0.075 (distant), 0.020 (2X) and 0.0167 (4X) expected substitutions per site. The sequences were simulated according to the JC69 substitution model, with root sequences of length 1000 nucleotides, and a rate of 1 indel per 25 substitutions. The indel length was drawn from the Poisson distribution with mean 1.7 nucleotide bases.

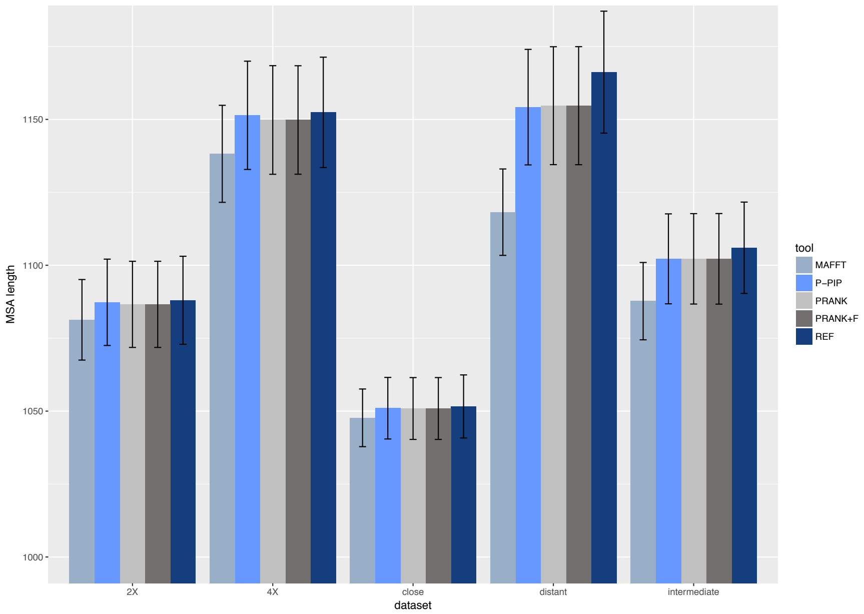

Figure S1 MSA length. The labels REF, PRNK, PRNK+F and P-PIP refer to REFERENCE alignments, PRANK, PRANK using the 'F' option and our Progressive-PIP algorithm, respectively. The values reported correspond to the mean MSA length over 250 simulations for each different evolutionary scenario, standard deviation in parentheses.

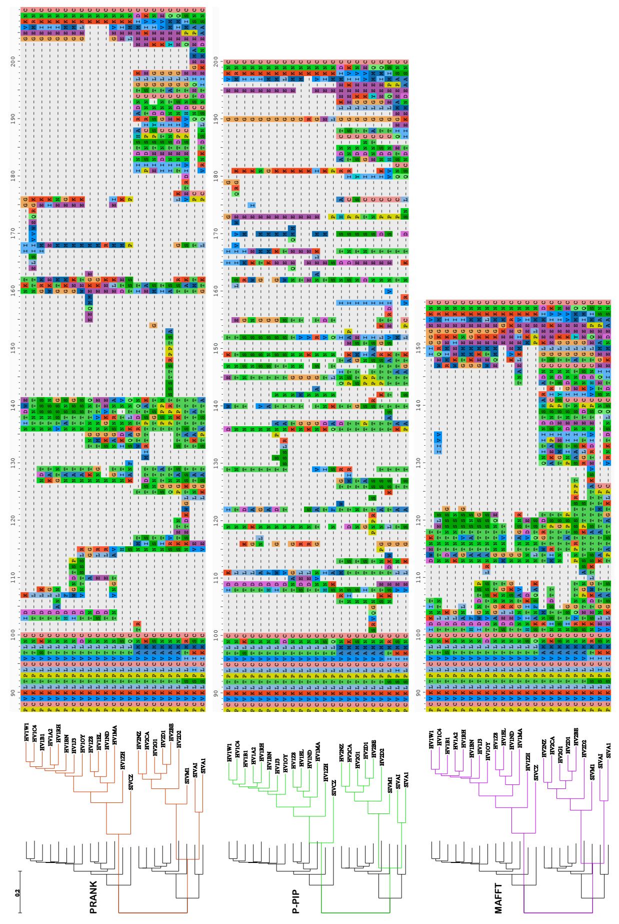

Figure S2 Magnification of Region 1 from the alignment of Figure 3.

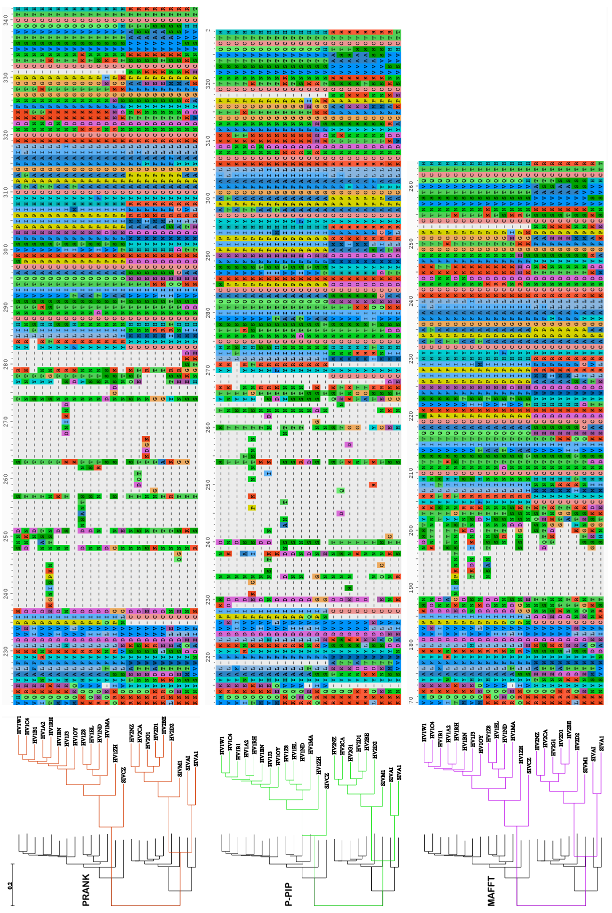

Figure S3 Magnification of Region 2 from the alignment of Figure 3.

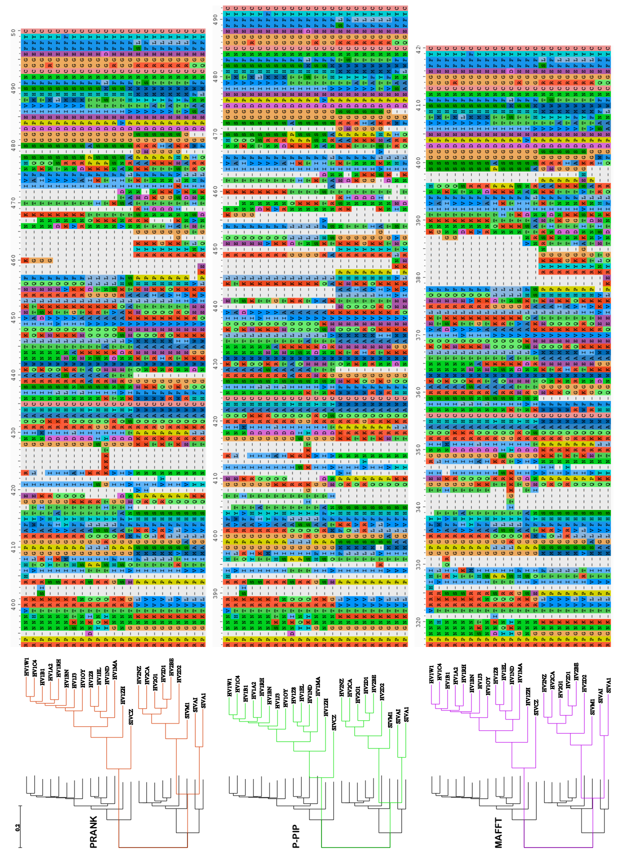

Figure S4 Magnification of Region 3 from the alignment of Figure 3.
